# Comparison the diagnostic value of serological and molecular methods for screening and detecting Chlamydia trachomatis in semen of infertile men: A cross-sectional study

**Published:** 2017-12

**Authors:** Amin Khoshakhlagh, Reza Salman Yazdi, Farah Taj Navab-Akbar, Azadeh Ghaheri, Shaghayegh Sadeghinia, Farid Dadkhah

**Affiliations:** 1 *Department of Microbiology, Islamic Azad University, Naein Branch, Isfahan, Iran.*; 2 *Department of Andrology, Reproductive Biomedicine Research Center, Royan Institute for Reproductive Biomedicine, ACECR, Tehran, Iran.*; 3 *Department of Microbiology and Virology, Isfahan University of Medical Sciences, Isfahan, Iran.*; 4 *Department of Epidemiology and Reproductive Health, Reproductive Epidemiology Research Center, Royan Institute for Reproductive Biomedicine, ACECR, Tehran, Iran.*; 5 *Department of Biomolecular and Biomedical Science, School of Health Life and Science, Glasgow Caledonian University, Glasgow, UK.*

**Keywords:** Chlamydia trachomatis, Male infertility, ELISA, PCR, Screening

## Abstract

**Background:**

*Chlamydia trachomatis* (CT) with damaging effects on sperm quality parameters can often cause infertility in men.

**Objective:**

The main objective of this study was to determine the diagnostic value of polymerase chain reaction (PCR) and enzyme linked immuno sorbent assay (ELISA) for screening and detecting CT in semen samples of infertile men.

**Materials and Methods:**

In this cross-sectional study, 465 men referring to the clinical laboratory of Royan Institute were chosen for primary screening and detection of the presence of CT. 93 samples were normozoospermia with normal sperm parameters i.e. sperm number, motility and morphology (Asymptomatic) and 372 had abnormal sperm parameters (Symptomatic) in semen analysis. ELISA test was performed as the screening test. Samples with optical density (OD) >0.200 were selected as the case and asymptomatic samples with OD <0.200 were selected as the control group for the confirmatory test. PCR assay was used to confirm the serological results.

**Results:**

In the case groups (n=62), 4 out of 32 symptomatic samples (12.5%), and 1 out of 30 asymptomatic samples (3.3%) revealed positive results in PCR. No PCR positive sample was observed in the control group (n=34). The final results revealed that considering OD >0.400 as the ELISA positive, the diagnostic value of CT-ELISA positive in symptomatic and asymptomatic infertile patients were 0.019 (7 of 372) and 0.021 (2 of 93), respectively. There was no relationship between the presence of CT infection and different sperm abnormalities.

**Conclusion:**

The anti-CT IgA ELISA test may be introduced as an appropriate tool for screening purpose in the seminal plasma to select suspicious samples for PCR confirmatory tests.

## Introduction

Male urogenital tract infection is one of the most important causes of male infertility (1). Genital tract infection and inflammation have been associated with 8-35% of male infertility worldwide (2). *Chlamydia trachomatis* (CT) is the most common etiological agent of sexually transmitted diseases after herpes simplex virus and human papilloma virus (3). CT is the most prevalent bacterial cause of sexually transmitted infections in the world and can result in severe genital disease. Over 90 million chlamydial infections are detected annually worldwide and various studies have estimated that there are four to five million new cases of chlamydial infection each year only in the USA (4, 5). However, the reported incidence rates of genital chlamydial infections in the population are likely an underestimate because of the highly asymptomatic nature of the pathogen. Approximately, 75% of infected women and 50% of infected men have asymptomatic urogenital infections, representing a huge population of untreated individuals who can transmit the organism (4).

CT requires tissue culture for in vitro growth, a technique which is technically challenging and time consuming, and facilities do in specialized research laboratories only. While cell culture is used to be the ‘‘gold standard’’ for the laboratory diagnosis of CT infections, this has been superseded by much more rapid and sensitive nucleic acid amplification tests (6), that their accuracy in detection of CT infections has been shown in several studies (7, 8). Although these procedures are accepted as reliable and confidential tests, they require extensive facilities and also they are expensive and time-consuming. In practice, there are several methods for detection of Chlamydia infection such as cell culture, enzyme-linked immunosorbent assay (ELISA), microimmunofluorescence, direct fluorescence antibody and molecular methods such as polymerase chain reaction (PCR) (9). 

Semen samples of infertile men are appeared to be a suitable source for tracking CT infection related infertility. Thus, the finding of a practical, rapid and cost-effective screening test that could be suitable for diagnostic purposes of CT infections in semen samples are demanded. 

The main objective of the present study was to determine and compare the diagnostic value of serological and molecular methods in screening and detecting CT infection in semen samples of infertile men referring to Royan Institute.

## Materials and methods

**Study population**

In this cross-sectional study, 465 men referred to Royan Institute, Tehran, Iran participated after providing written informed consent. All men were undergoing semen analysis as a part of work-up for infertility investigations. There were no age restrictions for inclusion in the study. All men with the history of chemotherapy or radiotherapy to the groin were excluded.

**Study groups**

The participants (465 individuals) were chosen for primary screening and detection of the presence of CT among which 93 samples were normozoospermia with normal sperm parameters i.e. sperm concentration, motility and morphology (Asymptomatic group) and other 372 had abnormal sperm parameters (Symptomatic group) in semen analysis (including Oligozoospermia, Asthenozoospermia, Teratozoospermia or leukocytospermia).

**Semen analysis**

Immediately following semen production, the sample was placed in an incubator and allowed to liquefy at 37^o^C for up to 30 min before the analysis commenced. Semen analysis was performed according to the World Health Organization guidelines (10). For this purpose, sperm concentration and motility were assessed by CASA (Computer-Aided Sperm Analysis) method by using Videotest Image Analysis-Sperm 2.1 (Videotest, Moscow, Russia), sperm morphology was measured by manual microscopic evaluation after Papanicolaou staining, and leukocyte count was done after peroxidase staining.

**ELISA test**

ELISA test by using commercial Vircell kit (Catalogue no. A1017, Spain) was performed as the screening test to trace the presence of anti-CT IgA in patients' seminal plasma. In this assay, they have used Complexes of Outer Membrane Proteins of CT, free from Lipopolysaccharide which is responsible for most cross-reactions with other Chlamydia species. 62 samples (32 symptomatic and 30 asymptomatic) with higher results in ELISA (Optical density (OD) >0.200) were selected as the case group and 34 asymptomatic samples with negative results (OD <0.200) were randomly selected as the control group for the confirmatory test.

**DNA extraction**

Sperms' DNA was extracted by using Qiagen extraction kit (QIAamp DNA Mini Kit, Cat. no. 51306, Qiagen, Germany) according to the manufacturer’s instructions. In order to confirm the presence of CT genome, DNA amplification was performed by using specific primers. The quality and quantity of extracted DNA were assessed with a NanoDrop 1000 spectrophotometer (Thermo Fisher Scientific) and typically had an A260/A280 OD ratio of 1.7-1.9. Extracted DNA was stored at -80^o^C until used.

**Nested PCR**

Nucleic acid amplification tests, which include nested PCR, are more typically used for screening and diagnosing chlamydial infections. The plasmid target DNA is present only in CT and at 7-10 copies per genome; therefore, targeting it with PCR increases the sensitivity and specificity of the test (11-13).

Nested PCR consisted of two rounds of amplifications using two sets of primers, and it was carried out as previously described (14). Detection of CT was done using initial primers T1/T2 for the cryptic plasmid of CT and secondary primers (T3/T4) internal to the initial primers (Table I) (14-17). The primers were synthesized by Pishgaman Inc. (Tehran, Iran).

**Figure 1:**
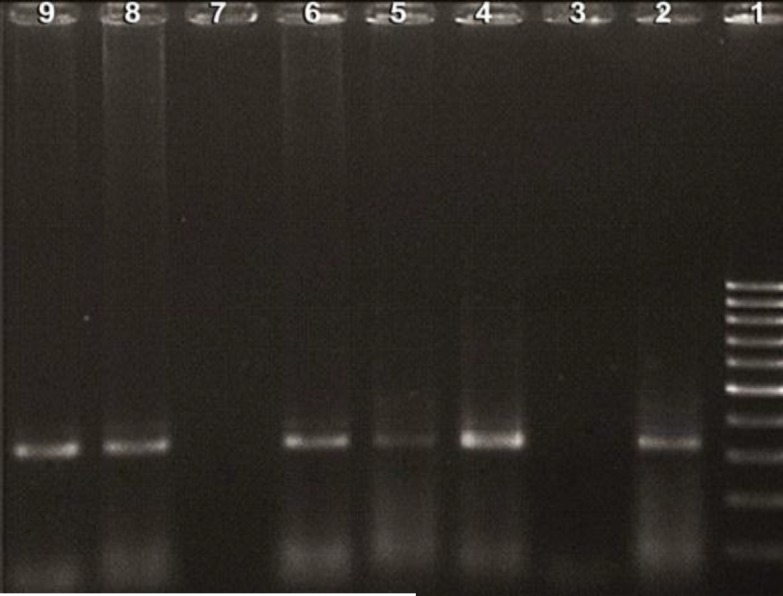
Agarose gel electrophoresis analysis of CT PCR products.

PCR was performed with a final volume of 25μl containing final concentrations of 50mM KCl, 10mM Tris-HCl (pH=8.3), 1.5mM MgCl2, 200μM each deoxynucleoside triphosphate (dATP, dTTP, dGTP, and dCTP), 25pmol of each outer primer, and 1 U of Taq DNA polymerase (Fermentas, Lithuania). The thermal condition consisted of initial denaturation at 94^o^C for 7 min followed by 35 cycles of the denaturation step at 94^o^C for 1 min, an annealing step at 55^o^C for 1 min and the elongation step at 72^o^C for 1 min. In the second round PCR, 1μl product from the first PCR step and 0.4 μM each T3/T4 inner primer pair was added to the final volume of 25 μl. PCR conditions were as described above. The expected PCR product of 320bp was examined on a 0.8% agarose gel after ethidium bromide staining and was confirmed by sequencing and comparison of the amplicon to GenBank accession no. 144462. CT serovar LGV1 DNA was used as a positive control, and distilled deionized water in place of DNA template was used as a PCR negative control to demonstrate whether there was any contamination with CT DNA. A DNA ladder (100bp) was also run simultaneously to confirm the size of the amplified product (Figure 1).

**Ethical consideration**

The study was carried out from June 2015 to August 2015 and approved by the Research and Medical Ethics Committee of the Royan Institute (REC.1395.72). The aim and objective of the study and the confidentiality of the data were explained verbally to the infertile men by urologist prior to their participation. Written informed consent was obtained from all participants before completing the measures.

**Statistical analysis**

Continuous variables were expressed as mean±SD and categorical variables as number (%). Means of the groups were compared using one-way ANOVA and pair-wise multiple comparisons were done using Tukey test. When data were not normally distributed the non-parametric test of Kruskal-Wallis was used instead of ANOVA. The statistical analysis was performed using Statistical Package for the Social Sciences, version 16.0 (SPSS Inc, Chicago, Illinois, USA), and a p<0.05 was considered significant.

**Table I:** Primer sequences for *Chlamydia trachomatis*

**Name**	**Sequence**	**Amplicon size (bps)**
T1	5/-GGACAAATCGTATCTCGG-3/	517
T2	5/_GAAACCAACTCTACGCTG-3/	
T3	5/-ATTAACCCTCACTAAAGGGA-3/	320
T4	5/-GCCATGTCTATAGCTAAAGC-3/	

## Results

The results of screening ELISA tests are summarized in table II. 62 out of 465 samples had OD >0.200 in ELISA screening test and were selected as the case groups for molecular assay. 34 asymptomatic samples with OD <0.200 in ELISA test were randomly selected as the control group for PCR, as well.

The results of confirmatory PCR tests are summarized in table III. In the case groups, 4 out of 32 symptomatic samples (12.5%), and 1 out of 30 asymptomatic samples (3.3%) showed positive results in PCR. No PCR positive sample was observed in control group. In addition, all positive samples in PCR test had OD>0.400 in ELISA test. Therefore, considering OD >0.400 as ELISA positive, the relative frequency of ELISA positive in symptomatic and asymptomatic groups were 0.019 (7 of 372) and 0.021 (2 of 93), respectively, and the prevalence of ELISA positive (OD >0.400) was 1.9% (95% CI 1.0-3.7) (18).

The results of semen parameters in study groups are summarized and compared in table IV. There was no significant difference regarding the age between symptomatic (38.53±8.451, range: 25-69 yr) and asymptomatic (36.07±8.366, range: 24-57 yr) groups (p=0.253). There was a significant difference between sperm volume of the control group and the case groups (p=0.002), while no significant difference between symptomatic and asymptomatic groups (p=0.447). Regarding the sperm concentration, there was a significant difference between the average of sperm concentration of the symptomatic group against two other groups (p<0.001), whereas the sperm concentration between the asymptomatic and control groups had no significant difference (p=0.747). 

Similarly, for sperm motility and morphology, there were significant differences between the averages of sperm motility and morphology of the symptomatic group against both the other groups (p<0.001), whereas the averages of sperm motility and morphology between the asymptomatic and control groups had no significant difference (p=0.213 and p=0.401, respectively). 15 out of 372 symptomatic samples had leukocytospermia (white blood cell count ≥1.0 million/ml) and only one of them was PCR positive; the all other CT-PCR positive samples were negative for white blood cell.

**Table II:** The results of ELISA test according to OD of samples

**Patient’s classification**	**Number**	**Cut-off OD= 0.200**		**Cut-off OD= 0.400**	
		**OD> 0.200**	**OD< 0.200**	**OD> 0.400**	**OD< 0.400**
Symptomatic	372 (80)	32 (8.6)	340 (91.4)	7 (1.9)	365 (98.1)
Asymptomatic	93 (20)	30 (32.3)	63 (67.7)	2 (2.2)	91 (97.8)
Total	465 (100)	62 (13)	403 (87)	9 (1.9)	456 (98.1)

Data are shown as Number (%)

OD: Optical density

**Table III:** Frequency of CT infection in study groups resulted by means of PCR

**CT**	**Case groups**		**Control group**
	**Symptomatic**	**Asymptomatic**	
Positive	4 (12.5)	1 (3.3)	0 (0)
Negative	28 (87.5)	29 (96.7)	34 (100)

Data are shown as number (%)

CT: *Chlamydia trachomatis*

**Table IV:** The results of semen parameters in study groups

**Semen parameters**	**Case groups**		**Control group (n= 34)**	**p-value**
	**Symptomatic (n= 32)**	**Asymptomatic (n= 30)**		
Semen volume (ml)	2.84 ± 1.46^a^	3.12 ± 1.41^ab^	4.07 ± 1.41^c^	0.002
Sperm concentration (×10^6^/ml)	39.64 ± 30.43^a^	85.57 ± 32.84^bc^	79.65 ± 33.59^c^	<0.001
Sperm motility (%)	28.21 ± 14.80 ^a^	63.35 ± 11.02^bc^	57.99 ± 11.70^c^	<0.001
Sperm morphology (%)	2.53 ± 2.02 ^a^	5.93 ± 1.66^bc^	6.32 ± 1.80^c^	<0.001
WBC in semen (×10^6^/ml)	0 (0-1.7) ^a^	0 (0-0.8)^b^	0 (0-0)^c^	<0.001

WBC: White blood cell; Data are presented as Mean±SD or Median (min-max) when appropriate.

(abc) Different letters indicate significantly different means (Tukey test of the one-way ANOVA).

Lane 1: 100 bp DNA ladder.

Lane 2: Positive control (320bp).

Lane 3: Negative control.

## Discussion

In this study, we were going to determine and compare the diagnostic value of serological and molecular methods in screening and detecting CT infection in semen samples of infertile men. Considering the fact that CT is an obligatory intracellular parasite and its presence in urethral epithelial cells can induce a secretory immune response by producing IgA, in our study we were planning to try on Anti-CT IgA ELISA kit as a screening tool for detection of CT in seminal plasma of infertile men. Since there was no comment in the kit instruction about the Cut-off value for the seminal plasma, outset we choose OD >0.200 as Cut-off value to select all the suspicious samples without any false-negative results. After confirming the results by PCR, the revision of ELISA output showed that we can set the OD >0.400 as a cut-off value for semen samples, and it can cover all the positive samples without any false-negative. In this way, the sensitivity and specificity of the assay were 100% (5 of 5) and 95.6% (87 of 91), respectively.

Male urogenital tract infections are discussed as one of the significant etiological factors for male's infertility worldwide (1). Infectious processes may lead to deterioration of spermatogenesis, impairment of sperm function or obstruction of the seminal tract (19). According to previous studies, the prevalence of CT genital infection in some developed countries ranges from 1-25%, considering sexual orientation, number of partners and socioeconomic status of patients (20, 21). Perhaps because of social conservatism in Iranians regarding free sexual attitudes, the prevalence of sexually transmitted diseases including CT genital infection may not be as high as in more-developed countries, but the results of several studies show that appropriate preventive strategies for CT should be considered in our country (22, 23).

A couple of previous studies in Tehran carried out on male patients attending sexually transmitted infection clinics for urethritis showed that 8.8% and 8.4% of those patients were infected with Chlamydia (22). By using cell culture method, Ghanaat and colleagues showed that 9.3% Iranian male patients with urethritis attending clinics in Mashhad were infected with Chlamydia (22). Sadrpour and co-workers in a survey at Avicenna Infertility Center had studied 120 men who had an abnormal semen analysis and reported that 23.3% of the samples were CT-PCR positive (24). Hamdad-Daoudi and colleagues reported that 2.7% of semen samples of asymptomatic male partners of infertile couples in France were PCR positive for CT (13). 

Günyeli and colleagues in a serological investigation of urethral and cervical samples with respect to the prevalence of Chlamydia and sperm parameters showed that no significant difference between the fertile and infertile groups in terms of both mentioned items (25). In our study, the prevalence of ELISA positive (OD >0.400) was 1.9% (95% CI 1.0-3.7), and 4 out of 32 (12.5%) symptomatic patients and 1 out of 30 (3.3%) asymptomatic patients were PCR-positive (18). The discrepancy in the prevalence of chlamydia infection between the current study and previous investigations may be caused by differences in culture, religious commitment such as avoiding sex outside marriage, different sample sizes and types, the time period of sampling, and diagnostic methods.

Regarding the sperm abnormalities, in the current study as expected, significant differences have observed between the averages of different sperm parameters (sperm concentration, motility, and morphology) of the symptomatic group against both asymptomatic and control groups, whereas those parameters between the asymptomatic and control groups had no significant difference.

In our study the percentages of CT-ELISA positive (OD >0.400) samples in symptomatic and asymptomatic groups (1.9% and 2.2%, respectively, Table II) were almost alike, hence it can be concluded that there was no relationship between the presence of CT infection and different sperm abnormalities (including sperm concentration, motility, and morphology). This result was in accordance with several studies (25-27). However, some other studies have shown that CT infection is associated with poorer semen quality (28, 29).

Our results indicated no significant difference regarding the age between symptomatic and asymptomatic groups, and all infected cases were in the range of 25-43 yr, whilst Golshani and colleagues showed that the highest number of infected cases belonged to the range of 20-30 yr (30). Our results illustrated that there was a significant difference between sperm volume of the control group and the case groups, while no significant difference between symptomatic and asymptomatic groups. Likewise, Gdoura *et al* reported no significant difference between the mean values of the seminal volume of infected cases (26).

To observe an elevation in leukocyte numbers in the ejaculation of men with a bacterial infection is not a surprise. However, in this survey only one CT DNA-PCR positive sample was leukocytospermic, and conversely, there were 4 CT DNA-PCR positive samples with no elevated numbers of leukocytes in the semen. Therefore, our results showed that there was no relationship between CT infection and the leukocytospermia. The presence of leukocytes in CT negative specimens may be explained by the presence of other genitourinary tract infections that were not screened in this study. The lack of concordance between leukocyte numbers and CT infection has been observed in some other studies and there is often a poor link between CT infection and the number of leukocytes in an ejaculation (14).

According to previous studies, screening program will be cost-effective if the prevalence of CT among men and women is at least 6% (23). Since the incidence of CT in the current study was lower than 6%, screening of all infertile men by PCR is not cost-effective and affordable. Thus, in order to obtain more epidemiological information, low-cost techniques such as serological methods can be recommended. Although the number of PCR-positive samples was few in this study, we recommend that using Anti-CT IgA ELISA test by cut-off OD >0.400 for semen specimens might be a good choice for screening semen samples, with acceptable sensitivity and specificity.

In this context, it is suggested that studies with broader distribution and sampling time and larger sample size be performed to determine real prevalence of chlamydia infection and make a definite decision about screening programs. In addition, concurrent studies in other communities at risk and patients with clinical syndromes such as infertility, epididymitis, urethritis and prostatitis will be helpful to find national statistics

## Conclusion

*Chlamydia trachomatis* urogenital infection should be timely diagnosed and well treated, otherwise, it will lead to pelvic inflammatory disease and the other types of urogenital sequela in patients. The process of screening of infertile men with no clinical symptoms is inevitable and is taken into account as an integral part of sexually transmitted disease control programs. Our study demonstrated that the Anti-CT IgA ELISA test could be introduced as a suitable tool for screening purpose in seminal plasma of infertile men to distinguish more probable infected patients for PCR confirmatory tests.

## Note

***This article extracted from M.Sc. thesis. (Amin Khoshakhlagh)***
